# Methicillin-resistant–*Staphylococcus aureus* Hospitalizations, United States

**DOI:** 10.3201/eid1106.040831

**Published:** 2005-06

**Authors:** Matthew J. Kuehnert, Holly A. Hill, Benjamin A. Kupronis, Jerome I. Tokars, Steven L. Solomon, Daniel B. Jernigan

**Affiliations:** * Centers for Disease Control and Prevention, Atlanta, Georgia, USA

**Keywords:** antimicrobial resistance, MRSA, Staphylococcus aureus, ICD-9-CM

## Abstract

Methicillin-resistant *Staphylococcus aureus* (MRSA) is increasingly a cause of nosocomial and community-onset infection with unknown national scope and magnitude. We used the National Hospital Discharge Survey to calculate the number of US hospital discharges listing *S. aureus*–specific diagnoses, defined as those having at least 1 International Classification of Diseases (ICD)-9 code specific for *S. aureus* infection. The number of hospital discharges listing *S. aureus*-specific diagnoses was multiplied by the proportion of methicillin resistance for each corresponding infection site to determine the number of MRSA infections. From 1999 to 2000, an estimated 125,969 hospitalizations with a diagnosis of MRSA infection occurred annually, including 31,440 for septicemia, 29,823 for pneumonia, and 64,706 for other infections, accounting for 3.95 per 1,000 hospital discharges. The method used in our analysis may provide a simple way to assess trends of the magnitude of MRSA infection nationally.

*Staphylococcus aureus* is a major cause of infection in both healthcare and community settings. It is one of the most common causes of healthcare-associated infections reported to the National Nosocomial Infections Surveillance (NNIS) System, including ventilator-associated pneumonia, surgical site infection, and catheter-associated bloodstream infection (1). *S. aureus* is also a frequent cause of community-associated infections, particularly skin and soft tissue infections. Although most community-onset infections are treated in the outpatient setting, some invasive infections, including bacteremia, septic arthritis, toxic shock syndrome, osteomyelitis, and endocarditis, have devastating complications and may require hospitalization (2).

Antimicrobial resistance in *S. aureus* emerged soon after penicillin came into common use in the 1940s. During the next 2 decades, resistance of this pathogen to penicillin became widespread, followed by increasing resistance to the new semisynthetic penicillinase-resistant antimicrobial drugs (e.g., methicillin, oxacillin, nafcillin) (3). In the last 20 years, methicillin-resistant *S. aureus* (MRSA) has spread throughout the world in healthcare settings, leading to an increased reliance on vancomycin for empiric treatment (4). Recently, *S. aureus* resistance to vancomycin, the last commonly used antimicrobial drug to which this organism was considered uniformly susceptible, has emerged (5). In addition, serious MRSA infection has been increasingly reported in persons without identified predisposing risk, including recent healthcare exposure (6).

MRSA infections are thought to cause substantial illness and contribute to healthcare costs in the United States. However, published estimates vary widely and have been based on single-center or local data with limited applicability (4,7). Accurate estimates of the incidence of MRSA infection are essential to determine effects on health and healthcare expenditures. Since most patients with serious MRSA infections are hospitalized, we focused our estimate on hospitalized patients.

## Methods

The incidence of *S. aureus* infection was estimated from the number of hospitalizations with *S. aureus*–related discharge diagnoses in a national surveillance database. We used 1999 and 2000 public-use data from the National Hospital Discharge Survey (NHDS) to calculate the number of hospital discharges with at least 1 *S. aureus*–related discharge diagnosis. All acute-care hospitalizations, except infants whose hospital stay began at their own birth, were included. The NHDS is a nationally representative annual sample of discharge records from ≈475 nonfederal short-stay hospitals (8). The survey is based on a stratified, multistage probability design; the sampled hospital discharge records are weighted to produce national estimates. The database includes <7 principal discharge diagnoses. We identified *S. aureus*–related discharge diagnoses by using the International Classification of Diseases, Ninth Revision, Clinical Modification (ICD-9-CM) codes specific for *S. aureus* infection: 038.11 (*S. aureus* septicemia), 482.41 (*S. aureus* pneumonia), and 041.11 (*S. aureus* infection in conditions classified elsewhere or of unspecified site). A discharge record listing multiple *S. aureus*-related diagnoses was counted only once. Septicemia was preferentially included, followed by *S. aureus*–related pneumonia.

Next, the percentage of isolates resistant to oxacillin was determined. To simplify terminology, resistance to methicillin and oxacillin hereafter are used interchangeably. Oxacillin is used as a proxy for testing of susceptibility to all β-lactam antimicrobials, including methicillin. The Surveillance Network (TSN) Database-USA (Focus Technologies, Herndon, VA, USA) was the source of antimicrobial susceptibility testing results. TSN is a repository of quantitative and qualitative susceptibility results collected from >200 microbiology laboratories in the United States. These laboratories make up a nationally representative sample based on associated hospital bed size, patient population, and geographic region as determined by the US Bureau of the Census (9,10). Susceptibility testing of patient isolates is conducted on site by each participating laboratory as part of routine diagnostic testing; only isolates judged as clinically significant are included. Data are generated by using Food and Drug Administration–approved testing methods. *S. aureus* antimicrobial susceptibility to oxacillin was classified as susceptible, intermediate, or resistant according to NCCLS breakpoint criteria; we classified intermediate isolates as methicillin-susceptible *S. aureus* for purposes of this analysis. Data were stratified by site of infection, i.e., bloodstream, lung, and other sites. Duplicate isolates were removed if the initial and subsequent isolates were cultured within 30 days of each other.

The number of hospital discharges listing *S. aureus*–specific diagnoses was multiplied by the proportion of methicillin resistance at each corresponding infection site to determine the total number of MRSA infections. Infections also were stratified by geographic region and age. The frequency of primary diagnosis and the 10 most frequent secondary (all-listed) diagnoses were abstracted from hospitalizations that included *S. aureus*–specific diagnoses.

Results for the years 1999–2000 were determined by calculating data specific to each year and then averaging. Data on resistance rates were stratified first by region and then by age; for each stratification, a chi-square test was used to determine whether differences were significant. The Cochran-Armitage test, a nonparametric method, was used to determine the trend in MRSA hospitalization rate by age category.

The effects of region and age on the incidence rate of MRSA were assessed by calculating relative rates and their associated 95% confidence intervals, with the lowest rates designated as comparison groups. Since the rate of *S. aureus* and the MRSA proportion were estimated separately and then multiplied to obtain the MRSA hospitalization rate, the variance of the MRSA rate was calculated by using the delta method (11). The variance of the methicillin resistance proportions was determined under the assumption that the antimicrobial susceptibility data reflected those that would have been derived from a random sample of all *S. aureus* isolates in the United States in that time period. Variance estimates were calculated using SUDAAN software (Research Triangle Institute, Research Triangle Park, NC, USA). For both *S. aureus* rates and methicillin resistance proportions, variances were estimated separately for 1999 and 2000, and the larger of the variance estimates was used in subsequent calculation of 95% confidence intervals for relative rates.

## Results

We estimate that 291,542 hospital discharges with *S. aureus* infection-related diagnoses occurred annually from 1999 to 2000 (Table 1). A diagnosis of *S. aureus* infection occurred in 9.13 of every 1,000 hospital discharges. The overall rate of methicillin resistance for all *S. aureus* infections was reported to be 43.2%. MRSA rates for septicemia, pneumonia, and other infections increased with patient age. An estimated 125,969 hospitalizations with 1 or more discharge diagnoses associated with MRSA infection occurred annually, accounting for 3.95 of every 1,000 hospital discharges. For all sites, most diagnoses occurred in persons >65 years of age.

**Table 1:** *Staphylococcus aureus*–related discharge diagnoses, United States, 1999–2000, by patient age and infection site*

Discharge diagnosis	Age (y)				
	<14	15–44	45–64	>65	Total†
*S. aureus* septicemias	2,918	12,272	20,028	38,948	74,166
Proportion of methicillin-resistant isolates from blood culture	0.144	0.317	0.392	0.495	0.424
MRSA septicemias	420	3,890	7,851	19,279	31,440
*S. aureus* pneumonias	2,328	5,582	6,926	41,427	56,263
Proportion of methicillin-resistant isolates from lower respiratory culture	0.195	0.333	0.467	0.586	0.530
MRSA pneumonias	454	1,859	3,234	24,276	29,823
Other *S. aureus* infections	14,290	39,222	40,496	67,105	161,113
Proportion of methicillin-resistant isolates from other culture sites	0.160	0.279	0.378	0.539	0.402
Other MRSA infections	2,286	10,943	15,307	36,170	64,706

*MRSA, methicillin-resistant *S. aureus*. †Due to rounding of methicillin-resistant proportions, total MRSA infections may differ slightly when estimates are calculated across category groups by row (i.e., age) compared with column (i.e., infection site).

In hospitalizations in which *S. aureus* septicemia and pneumonia were listed as discharge diagnoses, these conditions were primary diagnoses in 34.3% and 49.3% of discharges, respectively. For *S. aureus* infection in conditions classified elsewhere and in an unspecified site, a diagnosis intended only for secondary listing, the most frequent primary diagnoses were postoperative (e.g., wound) infection (10.1%), cellulitis or abscess (9.9%), infection from an implanted device or graft (7.3%), and urinary tract infection (3.6%).

The largest proportion of *S. aureus*–related discharge diagnoses occurred in patients from the South, followed by the Midwest, Northeast, and West (Table 2). For both, the rate of *S. aureus* discharge diagnoses and methicillin resistance proportion, significant differences were seen by geographic region. *S. aureus* discharge diagnoses were significantly higher for the South than the Northeast, while for methicillin resistance proportion, the Northeast, Midwest, and South were significantly higher than the West (p<0.05 for all comparisons). The South had the highest MRSA hospitalization rate, reflecting both the *S. aureus* rate and methicillin resistance proportion, which was significantly higher than the MRSA rate estimated for the West (South vs. West, relative risk 1.57, 95% confidence interval 1.29–1.91).

**Table 2:** *Staphylococcus aureus*–related hospitalizations, United States, 1999–2000, by geographic region*

Region	Discharge diagnosis			
	*S. aureus* (%)	*S. aureus* rate†	MR (%)	MRSA rate†
West	17.4	9.04	31.4	2.84
Northeast	20.5	8.51	41.3	3.52
Midwest	22.6	9.06	43.5	3.94
South	39.5	9.58	46.5	4.45

*MR, methicillin resistant; MRSA, methicillin-resistant *S. aureus*. †Rate, hospitalizations with *S. aureus*– or MRSA–related discharge diagnoses per 1,000 discharges.

Most *S. aureus*–related discharge diagnoses occurred in patients >65 years of age. When *S. aureus* diagnoses by rate were examined, a bimodal distribution was seen, with highest rates occurring in children and the elderly (Table 3). Patients <14 and 15–44 years of age had higher MRSA hospitalization rates compared with patients 45–64 and >65 years of age (p<0.01). Overall, the MRSA rate increased with patient age (p<0.05 for trend).

**Table 3:** *Staphylococcus aureus*–related hospitalizations, United States, 1999–2000, by patient age*

Age (y)	Discharge diagnosis				
	*S. aureus* (%)	*S. aureus* rate†	MR (%)	MRSA rate†	MRSA RR (95% CI)
<14	6.7	8.08	16.2	1.31	Referent
15–44	19.6	5.69	29.3	1.67	1.2 (0.94–1.6)
45–64	23.1	9.74	39.1	3.81	2.9 (2.2–3.8)
>65	50.6	11.76	54.1	6.36	4.8 (3.7–6.2)

*MR, methicillin resistant; MRSA, methicillin-resistant *S. aureus*; RR, relative risk; CI, confidence interval. †Rate, hospitalizations with *S. aureus* or MRSA-related discharge diagnoses per 10,000 discharges.

## Discussion

Infectious diseases cause many hospitalizations each year in the United States; these diseases include syndromes commonly associated with *S. aureus*. In 1994, the rate of hospitalization for infectious disease was 15 per 1,000 US population, with a total of 4 million hospitalizations, including 1,480,000 pneumonias, 335,000 skin infections, and 302,000 septicemias; yearly rates for these disease syndromes were similar from 1999 to 2000 (12–14). Gram-positive organisms are an increasingly recognized cause of systemic infection, including sepsis (12,15). More than half of all sepsis cases are estimated to be caused by gram-positive organisms, including *S. aureus* (16). In the Calgary Health Region in Canada, the annual incidence of invasive *S. aureus* infection was estimated to be 28.4 cases per 100,000 population from 1999 to 2000, which is comparable with the rate of invasive pneumococcal disease and exceeds the rate of invasive streptococcal infection (17).

Drug resistance in *S. aureus*, including the emergence of MRSA in healthcare and community settings, is an increasingly reported event that makes treating serious infection difficult. Extrapolating from our estimates and those of Simonsen et al. (12), a rate of ≈47 diagnoses per 100,000 population, making up 3% of all infectious disease hospitalizations, were associated with laboratory-confirmed MRSA infection from 1999 to 2000, and ≈10% of septicemias were caused by MRSA.

Although the burden of MRSA infection has not been systematically estimated nationally, past estimates have been based on single-center or selected population-based studies in the United States. Based on ICD-9-CM data from the New York City metropolitan area, an estimated 1.0% of hospital discharges are associated with *S. aureus* infection, and 0.21% of discharges are estimated to be associated with MRSA (18). In 1995, based on extrapolation of hospital discharge data from NHDS and nosocomial infection data from the NNIS System, an estimated 206,504 *S. aureus* infections (0.58% of admissions) and 70,270 MRSA infections (0.20% of admissions) were acquired in the healthcare setting (Centers for Disease Control and Prevention, unpub. data). Our estimates for 1999 to 2000 are similar for *S. aureus* infections but are higher for MRSA.

Although ICD-9-CM coding accuracy for *S. aureus* infections has not been specifically examined, the accuracy of coding for sepsis from all causes has been reviewed, and has demonstrated a sensitivity >75% for any septicemia or bacteremia code and positive and negative predictive values >80% for the code specific for *Staphylococcus* spp. septicemia (ICD-9-CM 038) (16,19). However, the relationship between true *S. aureus* infections and ICD-9 discharge coding should be further assessed to validate this method as a tool for monitoring national trends.

We found associations between MRSA rate and both region and age. This finding is consistent with previously published data showing an association between age and both the incidence of invasive *S. aureus* infection and the rate of methicillin resistance (17,20). We also demonstrated a significant difference in MRSA discharge rates between the South and West. Although past microbiologic surveys also have reported higher rates of methicillin resistance in the South compared with other regions, the reasons for this variation are unclear (21,22). These differences may need to be assessed as community-associated MRSA infection becomes more common.

Our estimate is subject to a number of limitations that most likely underestimated hospitalizations associated with MRSA infection. First, *S. aureus* infections may not have been accurately represented by the ICD-9-CM discharge code; colonization may have been inadvertently included; and more likely, true infections may not have been identified, since these diagnoses require laboratory culture confirmation. Since only 7 principal diagnoses are included in NHDS, infections listed less prominently may have been excluded. Duplicate isolates were excluded when identified within 30 days of each other; thus, unusual scenarios, such as multiple infections during a hospitalization or infections present for >30 days, were not included. We were not able to distinguish between community- and healthcare-acquired infection. However, this analysis was designed to measure the overall incidence of disease associated with acute care hospitalization, regardless of acquisition site, and did not include disease managed in the outpatient setting. Although previously published region and age stratification groups were used, which reduces risk of bias, unmeasured confounders may have affected calculated trends. Finally, although both NHDS and TSN data aim to represent nationally representative samples based on similar factors, methods may have differed, which could have skewed our results. For all data used, institutional settings, such as long-term care or correctional facilities, were not included.

In summary, our estimates indicate that the national burden of serious MRSA disease is quantifiable and substantial. Measurement of trends in *S. aureus* disease, such as the increasing incidence of antimicrobial resistance associated with certain age groups and geographic regions, will have implications in the development of prevention programs, both in the healthcare and community settings. Our method provides a simple way to estimate trends of magnitude of hospitalization associated with *S. aureus* infection in the United States and could complement methods currently in place for national surveillance.
