# Correction, Vol. 11, No. 6

**DOI:** 10.3201/eid1209.C21209

**Published:** 2006-09

**Keywords:** correction, erratum

In “Methicillin-resistant–*Staphylococcus aureus* Hospitalizations," by Matthew J Kuehnert et al., an error occurred. In Table 3, columns 3 and 5, the rates shown for hospitalization with *S. aureus* and MRSA-related discharge diagnoses were per 10,000 discharges, rather than per 1,000 discharges, as indicated.

The corrected table appears in the updated article at http://www.cdc.gov/eid/11/6/04-0831-t3.htm

We regret any confusion this error may have caused.

